# Domestic violence and social policy formation in Palestine: an explanatory sequential mixed-methods study

**DOI:** 10.3389/fsoc.2025.1730882

**Published:** 2026-01-06

**Authors:** Abdalrahim Shobaki, Noura Al-Shafai

**Affiliations:** 1 Department of Political Science, Faculty of Law and Political Sciences, An-Najah National University, Nablus, Palestine; 2 Faculty of Graduate Studies, An-Najah National University Libraries, Nablus, Palestine

**Keywords:** domestic violence, social policy, gender, Palestine, family protection, policy implementation

## Abstract

This study examines the relationship between domestic violence and the development and implementation of social policies in Palestine, focusing on the perceptions of officers in the Family and Juvenile Protection Department (FJPD). Adopting an explanatory sequential mixed-methods design, quantitative data were collected from 169 officers across the West Bank and Gaza through structured questionnaires, followed by 15 semi-structured interviews that provided qualitative depth and context. Findings reveal that domestic violence remains pervasive and multifaceted, with psychological and economic abuse identified as the most common forms. Statistical analyses indicate a strong negative correlation between the perceived prevalence of domestic violence and the perceived effectiveness of social protection policies (*r* = −0.61, *p* < 0.001). Regression results highlight institutional barriers (*β* = −0.47) and psychological violence (*β* = −0.31) as the most significant predictors undermining policy performance. Qualitative insights illuminate how entrenched patriarchal norms, limited institutional capacity, and practitioner burnout jointly constrain effective policy enforcement. Despite the existence of progressive legislative frameworks, implementation is weakened by fragmented coordination, insufficient resources, and the absence of a ratified Family Protection Law. The study contributes empirically and theoretically by linking micro-level patterns of violence with macro-level policy fragility. It underscores the need for integrated reforms legal ratification, institutional capacity-building, and cultural transformation to bridge the persistent gap between policy symbolism and substantive gender justice in Palestine.

## Introduction

Domestic violence is a widely acknowledged human rights violation worldwide and a significant societal and public health problem. It is a global issue that knows no cultural, economic or social boundaries and it impacts women, children and families everywhere. In patriarchal settings, domestic violence is often accepted as normal practice or justified through cultural and religious ideologies leading to the perpetuation of abuse and imbalances in power relations (Hamiwa, 2012; Randall, 2022).

In addition to the social dimensions, domestic violence has also become a crucial area of investigation for policy-makers and investigators on account of the role in influencing welfare and social protection programs. This article locates the experience of Palestinians within this wider global discourse, with a particular emphasis on how structural gender inequality and policy regimes overlap to influence responses to violence.

Domestic Violence in the Palestinian Context: This case of domestic violence against Naela is particularly layered and complex within a Palestinian context. Patriarchal cultural norms in conjunction with economic hardship, and prolonged political instability under occupation contribute to structural factors that make gender-based violence worse (Hassan, 2023). Palestinian women face multiple forms of oppression—that stemming from society-based gender hierarchies as well as political structures that restrict access to justice and resources. As a result, domestic violence cannot be understood simply as a problem of individual behavior or family dynamics; it is rather an expression of material inequalities inscribed in cultural, economic and political arrangements.

The most recent figures prepared by Palestinian Central Bureau of Statistics (PCBS) (2019, 2024), suggest that nearly six out of 10 women have been subjected to one or more types of violence: psychological, physical, sexual and economic. Policy changes and the development of family protection units in the police force have done little to ensure that this happens consistently, or with adequate resources. The disparity between lawmaking and law enforcement underscores a key policy challenge: even if legal tools are in place, institutional and cultural constraints hamper their effective attainment.

Social policies in Palestine have developed historically under both external constraints and changing political conditions. Following the formation of the Palestinian Authority in 1994, policy oriented toward gender and social welfare has been shaped by international conventions, donor directives, and development plans at a national level. Nonetheless, the continued breakdown of government and a scarcity of resources have restricted the utility of such interventions. Knowing and acknowledging how such violence structures, limits and perhaps even remakes the making of social policy is crucial to the future of institutional response (UNFPA, 2022; Ministry of Social Development, 2023).

From theory, domestic violence can be viewed through numerous theories. According to the social learning theory, violence by one generation would predict violence among posterity (Bandura, 1977). Feminist theory emphasizes the structural power differential between men and women as central to the existence of gender-based violence (Hamiwa, 2012). Theories of frustration–aggression and institutional describe the origins of violence as a consequence of socioeconomic deprivation, and weakened governing institutions (Hassan, 2023; UNFPA, 2022). Amidst Palestinian sociopolitical context, the unfavorable economic conditions of patriarchy and a protracted political conflict intersect to reproduce patterns of domination and submission which serve to legitimize spousal violence.

It is these theoretical insights that the current study seeks to develop with regard to the interrelationship of domestic violence and processes of social policy-making in Palestine. It does so, in particular, by focusing on the attitudes and beliefs of those professionals involved with carrying out state interventions aimed at preventing family violence: the staffers working in the Family and Juvenile Protection Department. Their views present exclusive empirical data about the practical difficulties of implementing policies, coordinating institutions and societal opposition.

This article endeavors to add to the burgeoning literature on policy relating to domestic violence in Palestine, through merging institutional with cultural analyses in an explanatory sequential mixed method design. By connecting day-to-day work in the RDF with larger policy narratives, it provides an explanation of how gender, governance and discourse intersect in dictating responses to DV. By doing so, the article demonstrates how legislative silences and social resistance work together to show that reforming the law will only be effective if it is accompanied by changes in the way rape is perceived.

### Research problem and significance

Although there is a vast body of work on domestic violence in Arab communities, only a few works have explicitly focused on the co-evolutional relationship between domestic violence and social policy making process in Palestine. Current studies have mainly focused on prevalence rates, psychosocial effects for survivors and the legal system, thus failing to take into account how policy efficacy is perceived by frontline workers and what are the socio-political boundaries they face (UN Women, 2023; UNFPA, 2022). We make a contribution to this hollow by utilizing survivors’ experiences of violence to macroscopic policy forces within Palestinian society.

To situate these challenges, it is important to get a sense of the legislative and institutional framework of family protection in Palestine. Under the Palestinian Civil Police, The Family and Juvenile Protection Department (FJPD) cooperates with the Ministry of Social Development and NGOs. However, gaps remain in legal mechanisms, which are a byproduct of the non-ratification of a Family Protection Law notably drafted since the beginning of the 2000s and perpetuates legislative vacuum concerning institutional liabilities and coordination on domestic violence (Ministry of Social Development, 2017, 2023; UN Women, 2024a,b). The proposed legislation, which seeks to create a coherent protection protocol, referral pathways and support for survivors of SV, is kept under the carpet due to socio-political polarization and backlash from conservative quarters. This institutional void has forced implementing agencies to resort to ad-hoc discretion and patchy coordination, further undermining the coherence of policy.

### Objectives of the study

The following are the objectives of the study:

1. To assess the prevalence and perceived forms of domestic violence within the Palestinian social context.
2. To examine the impact of domestic violence on the formation and development of social policies addressing family protection.
3. To determine demographic and professional attributes that affect the perceptions of the practitioners on the potential of the policy to be effective.
4. To point out structural, institutional and cultural obstacles which hinder the adoption of protective policies.
5. To propose policy recommendations for strengthening family protection systems and enhancing inter-agency collaboration.

### Research questions

The research is guided by the following key questions:

- How do practitioners perceive the relationship between domestic violence and the formulation or implementation of social protection policies in Palestine?
- What are the perceptions of officers working in the Family and Juvenile Protection Department regarding the adequacy of current policies?
- Are there statistically significant differences in perceptions based on gender, age, education, income, or geographic location?
- What institutional or socio-cultural factors constrain the effective application of protection policies?

### Hypotheses

According to the theoretical framework and the existing literature, the study hypotheses are:

1. Practitioners’ perceptions indicate a significant association between domestic violence and the formulation and implementation of social protection policies in Palestine.
2. No statistically significant differences exist in practitioners’ perceptions based on demographic variables such as gender, age, education, income, or residence.

### Structure of the paper

This article follows theme, and not numbering. It begins with a discussion of current literature on domestic violence and social policy that locates the Palestinian context as part of larger local and global conversations. It then explains the research methods, including a mixed method design and data collection process. The next section sets out the main empirical findings and discusses them in the light of relevant policy frameworks and gender theory. The paper ends with a discussion of the policy implications and institutional challenges as well as recommendations for promoting gender-responsive protection systems in Palestine.

### Contribution to knowledge

This article adds to the rapidly growing interdisciplinary literature on gender, violence and social policy in conflict-affected societies (True, 2018; Abu-Lughod, 2013). Implying the use of a single theoretical approach, rather than viewing IPV as multi-dimensional phenomenon, which requires feminist, institutional and policy analysis (Hassan, 2023; UNFPA, 2022). Connecting practitioners lived experience with macro level governance systems helps to further understand how structural gender power relations and the institutional piece-meal, scatter gun approach informs implementation of BPFPs (Palestinian Working Women Society for Development (PWWSD), 2021; WCLAC (Women’s Centre for Legal Aid and Counselling), 2024).

Empirically, the article offers elusive context-specific empirical evidence from a least studied Middle Eastern setting about how political instability and socio-cultural resistance restrict policy implementation (UN Women, 2024a,b). It seeks to contribute, in theory, current debates by showing that there is a need for intersectional and culturally responsive approaches when designing protection frameworks in occupied contexts (True, 2018).

## Literature review

### Theoretical and conceptual perspectives on domestic violence

Domestic violence is increasingly understood not merely as interpersonal aggression but as a socio-political phenomenon shaped by gendered power relations, institutional weaknesses, and socio-cultural resistance to reform (True, 2018; UNFPA, 2022). Cultural scripts become a standard in order to dominate and oppress women legally or informally in patriarchal and war-touched settings (Hassan, 2023; Abu-Lughod, 2013). The existing paradigm includes intersectional, institutional, and policy-oriented models of feminism and aims at analyzing how violence is reproduced due to structural imbalance, government failure and reinforcement of discriminatory social practices (UN Women, 2024a,b).

The multidimensional method broadens the analytic gaze aspect of the conventional feminist explanations that restrict the discussion of the problem to two-dimensional hardships (patriarchy and male dominance). It instead situates domestic violence within the broader political economy of welfare and social protection systems, highlighting how unequal power relations intersect with institutional capacity and policy design (WCLAC (Women’s Centre for Legal Aid and Counselling), 2024; Palestinian Working Women Society for Development (PWWSD), 2021). In the Palestinian context, these frameworks are essential to understanding why family protection policies remain fragmented and inconsistently enforced despite ongoing reform initiatives and international support (UN Women, 2024a,b).

Ultimately, this approach recognizes domestic violence not merely as a personal or cultural problem but as an outcome of intersecting social, political, and institutional forces that shape both women’s vulnerability and the state’s responsiveness.

### Forms and prevalence of domestic violence

Domestic violence in Palestine represents a deep social wound that extends beyond individual behavior to reflect structural inequality, cultural control, and institutional fragility. Past studies defined its manifestations as physical, psychological, sexual, and economic abuse premised on the power structure of the family and community life (Dobash and Dobash, 1992; Heise, 1998). Such classical models came in handy in defining the phenomenon but they barely knew how tension, presence and absence of occupancy, and poverty gave rise to such violence in the Palestinian context.

Only recent analyses are beginning to uncover these deeper layers. According to a study by Hassan (2023) and Palestinian Working Women Society for Development (PWWSD) (2021), the state of controlling women and poverty and insecurity are only enhanced by lack of an exit to violence due to patriarchal ideas and the absence of legal regulation, omnipresent even now. As noted in the reports issued by UN Women (2024a,b) and WCLAC (Women’s Centre for Legal Aid and Counselling) (2024), protection services remain unequal and unsynchronized and dependent on donor-led efforts perceived to be fragmented in lieu of a national policy. This institutional vacuum creates space within which silence and fear can seep into the gap created by justice and accountability.

Physical and psychological violence are the most prevalent but still, more significant than the acts of harm. They are a day-to-day infallibility of strength, life, and honor in families that are affected by patriarchal values and lessened state rules (UNFPA, 2022; Ministry of Social Development, 2017). The failure to enact the Family Protection Law has left both victims and practitioners without a clear legal path, reinforcing what True (2018) calls the “policy-practice divide” in gender governance.

To speak of prevalence is, then, not to enumerate the cases, but to speak of endurance—the mute perseverance of women whose terror is the walls of the family. Recent observations of UN Women (2024a,b) reveal that a good part of survivors prefers informal reconciliation as people are afraid of being branded stigma, retaliated, or not believed. This reality reveals how domestic violence in Palestine is not only a private matter but a public mirror of social injustice and institutional weakness. It will restrict the interpretation of its forms without the listening to lived experiences, trying to confront the structural inequality, and transforming the policy into the practical protection.

### Theoretical perspectives on domestic violence

Theoretical approaches to domestic violence have evolved from single-dimensional explanations of male dominance to more complex understandings that integrate cultural, institutional, and political dimensions. The early feminists learned to leave the issue of patriarchy to be their target and contended that any domestic violence of a woman is a product of structural subordination of the female both in the family and in the community (Dobash and Dobash, 1992). These foundational perspectives remain crucial for understanding gendered power relations; however, they are insufficient to explain how broader socio-political and institutional contexts shape patterns of violence and protection in conflict-affected settings such as Palestine.

The contemporary models have expanded the debate. Intersectional and institutional approaches highlight how gender inequality interacts with class, religion, and political instability to reproduce vulnerability and restrict women’s access to justice (True, 2018). Postcolonial feminist thought offers another level, the placement of violence within the framework of occupation structures and governance that destroys men and women simultaneously through structural dependency and control over their own bodies (UNFPA, 2022; Hassan, 2023). Such methods are challenging the patriarchy as a fixed local culture and as a dynamic system which is mediated by the political economy and vulnerability of the state.

From a social policy perspective, domestic violence is also framed as a governance problem resulting from institutional fragmentation, laws incoherence and the absence of enforceable forms or means to receive protection (UN Women, 2024a,b; WCLAC (Women’s Centre for Legal Aid and Counselling), 2024). This perception is shared with great effect in the Palestinian context where also no legal reform has yet passed that gives Family Protection Law force and everyone knows state-institutional protection to be good for nothing. It’s only by combining feminist, institutional and intersectional analysis that we can start to get to grips with domestic violence as both a symptom and cause of structural inequality.

Finally, these theoretical advances shift the focus of the prism from condemning the activities of particular individuals or cultural orientations to identifying structural defects in policy and governance. They recommend a multi-tiered approach that situates domestic violence at the crossroads of gender, power and institutional responsibility a model particularly fitting for societies experiencing political disintegration and social upheaval as in Palestine.

### Contributing factors/determinants

Domestic violence in Palestine cannot be reduced to individual behavior or family dynamics. It emerges as a composite of structural, socioeconomic, cultural, and political reality, which constitutes daily life. These criteria are overlapping areas that signify the replications of unequal relations of powerfulness in the situations of institutional weakness, economic hardship, and protracted political irregularity. In this sense, domestic violence is best understood as part of the broader landscape of gender-based violence, yet it carries distinct features in Palestine formed by the intersection of occupation, patriarchy, and fragmented systems of governance.

Economic and political insecurities remain among the strongest forces sustaining gendered violence. Chronic unemployment, poverty, and dependency on external gratitude pose additional pressure on the population in the country and restrict the options of women (UNFPA, 2022; Palestinian Working Women Society for Development (PWWSD), 2021). This unstable or dislocated position of men as the economic providers weakens their position and frustration and loss of social status may be turned into violence to restore power (Chant and Sweetman, 2012; Hassan, 2023). This economic insecurity and a power relationship between men is still powerful in the general negotiation of family power relations in modern day life.

At the same time, integrated expectations into culture are powerful elements that have enabled the element of silence in abuse. Family honor, obedience and sacrifice do not typically motivate the woman to be vocal and seek justice (Hassan, 2023). In a majority of the cases, when domestic disputes arise community mediators or elders in the family come what is the solution other than directly going to court which is reconciliation. They are capable of maintaining social harmony and on the other hand, this kind of interventions perpetuates the belief that the safety of women is not as important as the social stability of the family (Dobash and Dobash, 1992; Heise, 1998).

Meanwhile, institutional formats continue to point to the more extensive political dissection of Palestinian rule. Despite the establishment of Family Protection Units and repeated reform efforts, the absence of a ratified Family Protection Law leaves the system without clear authority or consistency (WCLAC (Women’s Centre for Legal Aid and Counselling), 2024; UN Women, 2024a,b). The absence of divisions between the west bank and Gaza, overlapping of areas of mandate/powers and a poor enforcement are undermining prevention as well as protection. The resistance to changes in the law, particularly conservative and religious forms of such resistance, only complicates the process and acts as a source of the discrepancy between traditional values and human rights demands (True, 2018).

The political background increases these issues. The relentless occupation, limitation in movement as well as frequent conflicts all elevate the vulnerability of women and limit them to support provisions. The institutional disappointment makes victims find justice in informal institutions, such as tribal councils, religious leaders, and extended families that usually have no intention to serve justice to victims but the stability of the community (UNFPA, 2022; Hassan and Akroush, 2024).

Only with a comprehensive system that will be informed by feminist/institutional/and intersectional knowledge we will be able to understand these intertwining determinants. Feminist analysis lays bare the persistent hold of patriarchal ideologies; institutional lenses reveal how poor governance perpetuates impunity, and intersectional frameworks expose how class, displacement, political precarity inform gendered experiences (True, 2018: Palestinian Working Women Society for Development (PWWSD), 2021). Collectively, these outlooks provide a reminder that domestic violence in Palestine is not indeed a private or individual phenomenon; it’s a societal mirror revealing the deeper inequalities that order this course of Palestinian life.

### Institutional and policy responses to domestic violence

Institutional and policy responses to domestic violence in Palestine have developed gradually within a fragile and politically constrained environment. The efforts at constructing a uniform system of protection have typically come into conflict with the greater fact of occupation, broken rule and social resistance to reform. While public institutions have made significant strides in acknowledging domestic violence as a policy concern, implementation remains partial and inconsistent.

The institutional organization of the Palestinian Civil Police, which plays the leading role in the reaction to the cases of family violence, is the Family and Juvenile Protection Department (FJPD). Its establishment was an important milestone that reflected growing recognition of domestic violence as a public issue rather than a private matter. However, the inadequate resources, the overlapping jurisdiction, and the absence of one legal framework continue to influence the work of the Department (UN Women, 2024a,b; WCLAC (Women’s Centre for Legal Aid and Counselling), 2024). The FJPD is inclined to organize civilians, the Ministry of Social Development, and the institutions of the civil society on a case-by-case, rather than on institutionalized, ground.

A central component of this policy landscape is the long-debated Family Protection Law. The law was originally drafted in the first half of the 2000s and was intended to create a comprehensive framework on prevention, protection and prosecution. Even though it was later revised multiple times and promoted by its supporters, it is still not ratified on a large scale, partly because conservative political forces refer to the act as a threat to family cohesion and cultural norms (True, 2018; Randall, 2022). It is so since the non-ratification has resulted in control of front line officers and social workers being left without a defined legal background to utilize the protection measures or bring offenders, who commit them, to book (UNFPA, 2022).

The current reports on the situation and the policy demonstrate that the issue of inconsistencies between the formal agreements and their substantive implementations is in the deeper flaws of the institutions. Protection units typically lack trained personnel, safe sites, and survivor referrals (Palestinian Working Women Society for Development (PWWSD), 2021). In the meantime, the collaboration with health, education and justice areas is quite fragmented. Theoretically, this brings unequal treatment of cases and informal mediation, especially in less liberal areas, where a community leader and spiritual figure is more likely to intrude on informal networks.

The civil society organizations, or the institutions that concern themselves with the rights of the women, have filled up part of this institutional vacuum. The small capacity of the government has been supplemented by shelters, legal assistance and awareness efforts of organizations such as WCLAC, the Women in Technical Committee and the Palestinian Working Woman Society of Development (WCLAC (Women’s Centre for Legal Aid and Counselling), 2024; Palestinian Working Women Society for Development (PWWSD), 2021). Their work has also been crucial in lobbying for the Family Protection Law and pushing for gender-sensitive policy reforms. Their act, however, is usually constrained by the structural characteristics like reliance on donation, political fragmentation as well as the social resistance of feminist activism (Dobash and Dobash, 1992).

The fact that there is constant breakdown of politics between the WB and Gaza also makes responding to it hazy. The administrative jurisdictions and disparate legal systems have contributed to the imbalanced protection and application of policies. Protection units in Gaza have merely been limited in their field of activity by traditional and religious courts yet efforts in promoting a transition to legal reform in the West Bank have only been hampered by bureaucratic delays and political priorities (UN Women, 2024a,b; Ministry of Social Development, 2023).

Feminist and institutional paradigms can be used to explain these institutional issues theoretically. The impact of patriarchal power distribution on the development and inhibition of policies on protection is known within the frames of feminist analysis, and the frailty of governance and absence of policy cohesiveness within the post-colonial and post-war states are known in the institutional theory (True, 2018; Randall, 2022). Within this context, domestic violence policy in Palestine becomes not only a social welfare issue but also a reflection of broader struggles over authority, legitimacy, and social transformation.

In the end, the Palestinian case also shows that policy on domestic violence cannot be dissociated from broader relations or power between leadership and resistance. The failure to ratify the Family Protection law is symptomatic of the tension between formal equality and actual practice, amidst decentralized enforcement and cultural resistance. Such endeavors, however, are being shaped by the continued efforts of state actors, women’s organizations and international institutions and agencies committed to the continued development of a more informed/exclusive/rights-based protection service set for Palestinians (WCLAC (Women’s Centre for Legal Aid and Counselling), 2024; UN Women, 2024a,b).

### Policy responses to domestic violence

Co-ordinated policy frameworks that aim at prevention, protection and prosecution. Many states have adopted comprehensive domestic violence laws aligned with international conventions such as CEDAW (Convention on the Elimination of All Forms of Discrimination Against Women). The holistic models that integrate law enforcement efforts with those of the victim and educate the population are often referred to as the Organic Law 1/2004 of Spain and the Family Violence Initiative of Canada (Heise, 1998). But there is inequality in the Arab region in terms of policy responses. The Law 103-13 (2018) of Morocco was a significant development in policing, yet it did not have support mechanisms in place and was criticized because of the poor application (Human Rights Watch, 2018). Similarly, Jordan and Egypt have introduced family protection units and national hotlines, yet patriarchal legal interpretations and societal stigma continue to hinder effective justice (El Feki, 2017; UN Women, 2023).

The Palestinian situation has witnessed the policy and institutional responses in the weak political-social context which have changed with time. The Family and Juvenile Protection Department (FJPD) established under the Palestinian Civil Police in 2010 serves as the primary mechanism for handling cases of domestic violence. It also collaborates with the Ministry of Social Development and different non-governmental organizations (NGOs) which provide psychosocial assistance and advocacy (Palestinian Police, 2013). However, there are still certain structural problems: underreporting, the absence of shelters, the shortage of female officers, inadequate inter-agency collaboration, etc. (Ministry of Social Development, 2023; WCLAC (Women’s Centre for Legal Aid and Counselling), 2024).

A central element of the national response is the draft Family Protection Law, which has remained unratified for nearly two decades. It has led to a regulatory gap that enhances institutional impunity and restrains protection of survivors (UN Women, 2024a,b; WCLAC (Women’s Centre for Legal Aid and Counselling), 2024). Local and international interest have pointed out the fact that the law is a crucial step toward establishing a unified and enforceable body of security. Palestinian Working Women Society for Development (PWWSD) (2021) noted that this additional delay during the ratification proves the sociocultural resistance to amend the law in its application has long existed. In the context of the ideological disparities in the civilization, one may also observe that the parliament debates on the draft law are distinguished by the fact of guilty feelings on both sides when on the one hand, the women rights groups and civil organizations are encouraging the government to begin applying the draft law and on the other hand, the conservative and religious groups will be inclined to discuss the draft law as the opposition to the culturally held values (Al-Araby Al-Jadeed, 2020).

This tension between modern legal reform and traditional social norms highlights the complex relationship between gender, law, and politics in Palestine. The UN Women Beijing +30 Report—State of Palestine (2024) reaffirmed that combating violence against women has been among the state’s stated priorities, yet progress remains constrained by reliance on voluntary coordination rather than binding legislation. Besides, other NGOs such as the PWWSD and the Women Affairs Technical Committee (WATC) are active in advocacy, legal sensitization, and information-gathering, but typically depend on the international donor funds rather than the state budgets (UNFPA, 2015; UN Women, 2024a,b).

Ultimately, the challenges facing domestic violence policy in Palestine can be grouped into three overlapping dimensions.

Firstly, the attitude to being a man and a dismissal of victims supports cultural resistance and makes reporting and justifying abuse a legitimate action, which remains present (Hassan, 2023; WCLAC (Women’s Centre for Legal Aid and Counselling), 2024). Second, the institutional constraints are caused by the unresponsiveness of the protection system and unfamiliarity with inter-agency coordination, resources, and professional training (Palestinian Working Women Society for Development (PWWSD), 2021).

Third, the disintegrated political structures between the West Bank and Gaza as well as the external influences of occupation weaken national structure and policy implementation (Ministry of Social Development, 2017; UN Women, 2024a,b).

These interlocking barriers demonstrate that effective policy reform in Palestine cannot be confined to legislation alone. It requires a parallel transformation in cultural attitudes, institutional capacity, and political will to ensure that protection from domestic violence becomes a lived reality rather than a rhetorical commitment.

It is on this contextual background that the analysis of institutional practice in the next section of the methodology will be conducted based on it.

### Gaps in the literature

The collection of empirical work on domestic violence in Palestine has grown over the past two decades, but a few important voids persist that impede theory building and policy formation. A great deal of the existing literature is concerned with prevalence rates, risk factors or psychosocial effects of abuse (Palestinian Central Bureau of Statistics (PCBS), 2019; UNFPA, 2015). These are vitally important studies for understanding the extent of the problem, yet they rarely examine how domestic violence intersects with larger social policies, governance and institutional reform. The disjuncture of gender-based violence with social policy analysis also left a void in our understandings of how violence constitutes while constituted by the functioning of welfare and protection systems within Palestine (UN Women, 2023; WCLAC (Women’s Centre for Legal Aid and Counselling), 2024).

Our second major limitation is the absence of the practitioners’ voice in the literature. Very little empirical research has direct working access to front line officers, social workers or policy makers who are involved in delivering protection structures. Their perceptions matter for uncovering obstacles and bottlenecks at the operational level, and differences between what is legal or policy law (Ministry of Social Development, 2023; Palestinian Working Women Society for Development (PWWSD), 2021). Second, the intersection of domestic violence with political conflict remains under-studied. As academics have pointed out, occupation-induced stress, displacement and sustained vulnerability influence family dynamics and exacerbate gender-based inequalities, but these elements are rarely dissected in analyses of domestic violence policy (Hassan, 2023; True, 2018; Randall, 2022).

A further area of limitation is the lack of longitudinal and evaluative studies. In Palestine and elsewhere, policy assessments concentrate on short-term interventions and donor-led programs with little systematic analysis of their long-term viability or utility (UNFPA, 2015; Ministry of Social Development, 2017). Furthermore, emerging frontiers such as digital media for awareness generation, reporting and advocacy mobilization are not well-researched within the Palestinian context (UN Women, 2024a,b). All these lacunae contribute to indicate the importance of research bridging the gap between micro-level experiences and policy dynamics at macro level.

This study aims to address these lacunae by adopting an integrated institutional, feminist, and intersectional approach to investigate cases where domestic violence interacts with the shaping of social protection regimes. By highlighting the experience of workers in the Family and Juvenile Protection Department, it provides time- and context-specific understanding of challenges to, and opportunities for policy implementation in Palestine.

### Summary

In general, the literature reviewed shows that domestic violence in Palestine is not simply a private or intimate phenomenon; rather, it is symptomatic of larger structural differences which find their roots within cultural, economic and political institutions. While there is global advancement in connecting gender-based violence to policy change, Palestinian scholarship on the issue remains piecemeal and narrow. In this line of research, the literature lack involve both practitioners’ voices and also a stronger integration between gender studies and social policy in general these limitations combined with the near absence of longitudinal evaluations have created knowledge gap that this study aims to bridge.

This study aims at filling this evidence-based, and policy and institutional analysis gap. It seeks to provide standard-based recommendations for enhancing protection standards and enabling gender-responsive social policies that are responsive to both the realities confronting frontline workers as well as the structural inhibitors hindering accountability in Palestine.

## Methodology

### Research design

This study was guided by an explanatory sequential mixed methods design, and used both quantitative and qualitative methodologies to investigate the impact of domestic violence on social policy processes in Palestine. The combined method was selected to generate such condition, in which the measurable tendencies appeared on the one hand and display on the other hand as well as dimensions of experience to allow analysis to be profound as well as stable as a statistical measure. This design combined both the proliferation of quantitative analysis and extensive subjection to the core of qualitative analysis to gain an overall understanding of the phenomenon (Creswell and Plano Clark, 2018; Flick, 2023).

The initial stage was an attempt to conduct a quantitative survey of the FJPD practitioners across the West Bank/Gaza Strip. This was followed by a qualitative period where semi-structured interviews on a sample subgroup were to be held to elaborate on the important statistical findings. This also enabled evidence triangulation, cross-validation of numeric trends as well as experiential accounts of the connection between policy perception and practice (Johnson and Onwuegbuzie, 2004; Bryman and Bell, 2023).

Questionnaires (structured) and interviews (semi-structured) were conducted in order to capture the views of officers’ and social workers of FJPD regard domestic violence policy effectiveness. The sample was diverse in content of gender, rank and geographic area to maximize generalizability. Quantitative information was assessed using descriptive and inferential statistics, and qualitative data were thematically coded to discover recurring institutionalized and cultural barriers (Hassan, 2023; Palestinian Working Women Society for Development (PWWSD), 2021).

This mixed-method approach serves to meet the direct aims of the study through relating their perspectives back to wider policy context. More specifically, the quantitative phase informed the first and second research questions focusing on policy adequacy and enforcement, while the qualitative phase provided a counter-point to these results based on participants experiences of living within this politically and socially constrained context.

All experimentation was performed in compliance with the ethical standards for studies involving human subjects. Consent was sought, voluntary to participate and confidentiality was observed in all stages of the research process (Bryman and Bell, 2023; UN Women, 2023; WCLAC (Women’s Centre for Legal Aid and Counselling), 2024).

### Research setting

The survey was carried out under the auspices of the Family and Juvenile Protection Department (FJPD) of the Palestinian Civil Police. This ministry is, for all purposes, the arm of government that deals with domestic violence and protection of children. It works with the Palestinian MoSD, Ministry of Women’s Affairs (MoWA), and UN and NGO-run shelters, counseling units, and legal aid services for victims of violence (Palestinian Police, 2013; WCLAC (Women’s Centre for Legal Aid and Counselling), 2024; UN Women, 2014).

This study possesses a distinct social and institutional setting of the Palestinian situation. The practitioners within the FJPD are operating under an amalgamation of contradicting forces of political cohesion, economic depression, and age-old cultural values, which continue to infuse the community approach to violence. The socio-political context of Palestine imposes an array of complex layers on the research environment, where structural violence is perpetuated through occupation, confinement and institutionally autonomous control (Hassan, 2023; True, 2018).

What field officers and social workers must daily leap over these are the barriers that they must overcome with grace and ingenuity, and can only overcome with grace and ingenuity with the aid of determination and innovation, part of which are by informal co-ordination and contacts within the community which are sufficient to fill in a protection law gap. Not only do the issues with bureaucracy weigh them down in their day-to-day work, but also the ethical and emotional burden of being in the position between the state policy and social tradition to regulate reproductive life (Ministry of Social Development, 2023; Palestinian Working Women Society for Development (PWWSD), 2021).

This context highlights the importance of understanding domestic violence policy as both professional practice and human struggle characterized by compassion, institutional vulnerability, and continuing aspirations for systemic change.

### Population and sampling

#### Target population

The study sample included all officers and employees of the Family and Juvenile Protection Division (FJPD) in the West Bank and the Gaza Strip. By 2024, there were some 320 officers and staff members who are formally registered and are spread across 12 administrative districts, both urban and rural areas (Palestinian Police, 2023). They are professionals focused on directly handling case management, social intervention, referral to legal and coordination with shelters and non-governmental organizations (NGOs).

Their work extends beyond administrative duties it involves daily negotiation between institutional mandates and community realities, often under the constraints of limited resources, gendered expectations, and public scrutiny (WCLAC (Women’s Centre for Legal Aid and Counselling), 2024).

#### Sampling procedure

A stratified random sampling approach was employed to ensure balanced representation across districts and gender. The reason is that the final sample composed of 170 officers acknowledged 53 percent of the total registered population. Following the process of data cleaning, 169 valid questionnaires that were left to undergo statistical analysis had a response rate of 99.4%.

The sample was purposely determined to facilitate the diversity of the department including both men and women operators with different education levels (diploma, bachelor, and master degrees) and working experience between 1 and 15 years. This variety made the comparative analysis more enriched in the demographic characteristics and predetermined the absence of biases in the points of view (Bryman and Bell, 2023).

For the qualitative phase, 15 participants were purposively selected from the survey respondents to provide in-depth reflections on institutional practices and policy implementation. Selection criteria included seniority, professional experience with domestic violence cases, and willingness to engage in open discussion. This purposive approach prioritized experiential insight over numerical representation, allowing for deeper understanding of the human and organizational challenges inherent in domestic violence intervention (Flick, 2023; UN Women, 2024a,b).

### Instrumentation

#### Questionnaire (quantitative instrument)

The study employed a structured questionnaire to gather quantitative data on practitioners’ perceptions of domestic violence and related policy mechanisms. It was developed by the tool accordingly to the former empirical tests (UNFPA, 2015; Palestinian Palestinian Police, 2023; Palestinian Working Women Society for Development (PWWSD), 2021) but changed with the assistance of the experts to become specific and cultural. The questionnaire contained 45 questions that were divided into four thematic areas:

- Demographic information covering age, gender, education, years of experience, and geographic location.
- Perceptions of domestic violence, consisting of 12 items assessing its prevalence and forms using a five-point Likert scale (1 = strongly disagree to 5 = strongly agree).
- Policy effectiveness, which consists of 18 items which assess legislation, institutional coordination and adequacy of resources.
- Implementation barriers 10 items: cultural, political, and logistical barriers.

These measures were measured using a five point Likert scale on which the consistency of internal consistency was assessed via the Cronbach alpha that was ranging between 0.82 and 0.91 that exhibited very high reliability (Gliem and Gliem, 2003). The questionnaire was intended to be distributed across the officers, yet a pilot study of questionnaires on a smaller group of the officers was performed to determine their clarity, cultural appropriateness, and technical precision (Bryman and Bell, 2023; Flick, 2023).

#### Qualitative instrument (interviews)

To accompany these quantitative findings, the study performed semi-structured interviews on 15 officers to elicit a portion of the human and institutional consequentialists in these quantitative patterns. The interviews further provided a better understanding of the difficulties experienced by practitioners, including countercultural opposition, resource constraints and psychological distress while working in family protection. The interview guide contained eight open ended questions which examined the experiences of the participants, perceived gaps in the policies, and recommendations on the reforms. All interviews, in the Arabic language, 45–60 min were transcribed and translated into English in order to perform thematic analysis.

This blended instrument approach enabled triangulation using statistical sources and narratives, thus yielding a more holistic and grounded sense of how domestic violence policies are being understood and practiced within Palestine’s complicated institutional context (Creswell and Plano Clark, 2018; UN Women, 2024a,b).

#### Validity and reliability

In order to make the study instruments robust and credible, a number of validation processes were used. Content validity was established through expert review: six specialists in social policy, gender studies, and criminology from *An-Najah National University* and *Birzeit University* evaluated the questionnaire for clarity, relevance, and cultural appropriateness. Their review was used to refreeze unclear items and improve the conceptual compatibility of the research aims.

The construct validity was established by the use of another method that is the *exploratory factor analysis* (EFA) that proved that there are four primary dimensions as confirmed by the theoretical model. The *Kaiser-Meyer-Olkin* (KMO) test of 0.88 and a significant test of *Bartletts test of Sphericity* (*p* < 0.001) were a good measure of adequate sampling and applicability to factor analysis.

Reliability was also tested by using a pilot test comprising of 20 participants who were not part of the main sample. *Cronbach’s alpha* values were above 0.80 in all areas indicating a high internal consistency and a high measurement stability (Gliem and Gliem, 2003; Bryman and Bell, 2023).

In the qualitative aspect, reliability was respectable according to the issues of credibility, dependability, confirmability within the categories of Lincoln and Guba (1985). These, were supported by triangulating and member checking the data, and having clear records of the analysis processes. Besides, the inclusion of a mixed-methods approach enhanced the validity of the study, cross-checking the quantitative and qualitative evidence (Flick, 2023; Creswell and Plano Clark, 2018).

#### Data collection procedures

The data collection was done in close collaboration with Family and Juvenile Protection Department (FJPD) between January and March 2025. The questionnaires were filled in electronically via the institutional email networks that are secure with the provision of printed copies in districts that lack internet connectivity to make them inclusive. All the respondents pre-participation received a clear explanation of the purpose of the study, voluntary nature of participation of respondents, and the firm confidentiality of the responses.

The quantitative stage was completed, and then the qualitative one was launched to further explore the emerging patterns. Semi-structured interviews with the chosen people sampled in the survey were scheduled and the researcher could enquire with them concerning certain issues institutional and cultural in preliminary findings. The interviews were either carried out privately in FJPD offices or through the safe online means (Zoom), based on the location and choice of participants.

Each of the sessions was recorded with the informed consent and later anonymized prior to the transcription and translation. It was supported by detailed field notes to maintain nonverbal and contextual information. All these procedures combined to make sure that data collection procedure was conducted with the highly ethical and methodological considerations in place, having the means to protect the participants and ensure the integrity of the data (Bryman, 2021; Flick, 2023; Creswell and Plano Clark, 2018).

### Data analysis

#### Quantitative analysis

The SPSS Version 28 was used to analyze quantitative data. Data analysis was done in three steps:

- Descriptive statistics (frequencies, means, and standard deviations) to describe the characteristics of the participants and responses to the items.
- Inferential statistics (ANOVA, t-tests and Pearson correlations) to test the connections between demographic variables and the perception of the efficacy of the policy in question.
- Regression analysis to assess the predictive effect of domestic violence prevalence on policy performance indicators.

The level of significance was established at *p* < 0.05. The associations were described by the effect sizes reported in terms of Cohen’s *d* and *η*^2^ to illustrate the strength of the associations (Cohen, 1988). The steps of applying the analytical procedures were used according to the best practices of an explanatory sequential mixed-methods study, which ensures rigor and interpretive depth (Bryman and Bell, 2023; Flick, 2023).

#### Qualitative analysis

The data was to be analyzed qualitatively through thematic analysis (Braun and Clarke, 2006) which enabled the patterns and meanings to be inductively uncovered as the participants relate their stories. Transcripts were coordinately read and coded to come up with repetitive ideas and phrases associated with institutional and social predicaments. There were three main themes realized:

- Cultural normalization of violence, how rules of patriarchy reproduce silence and justification of violence.
- The lock in of the institutional resources, a weakness of density in the personnel, training, and support of the logistics.
- Burnout and emotional stresses among practitioners with a special focus on the psychological tension of dealing with repetitive instances of violence.

NVivo 14 software helped to code and organize the themes. Data integration was done in the interpretation phase, with qualitative insights that expounded and provided the context of the quantitative findings, which is characteristic of the explanatory sequential design (Creswell and Plano Clark, 2018).

This analytic process ensured that statistical trends were grounded in human experience, providing both measurable and interpretive understanding of how domestic violence policies function in practice within Palestine’s institutional environment.

### Ethical considerations

Study approval was granted by the Institutional Review Board (IRB) at An-Najah National University (Ref. No. SP/25/2024). Participation was fully voluntary and anonymous. All individuals gave their informed consent before participation, and participants were informed that they could withdraw at any point without negative consequence. All data were securely encrypted, password protected and stored on servers, with any identifying information redacted during transcription to ensure anonymity.

Due to the sensitive nature of the research study topic, participants were given information about local psychological support services for counselling if they became distressed during the study. The researcher adhered to the APA ethic guidelines as set out in the Ethical Principles of Psychologists and Code of Conduct (American Psychological Association, 2017), ensuring respect, integrity and safeguarding at all times during research. These measures preserved both the moral and emotional protection of respondents as well as adding to the validity of results (Bryman and Bell, 2023; Flick, 2023).

### Weaknesses of the methodology

Although the mixed-methods, explanatory design facilitated triangulation and increased analytic depth, a number of shortcomings should be acknowledged. One, self-reported data are biased by the social desirability of responding in a certain manner; indeed, using an instrument to measure incidence or prevalence of at-risk status for domestic violence opens them up to questioning under-reporting (Tourangeau and Yan, 2007). Second, logistical challenges and occasional political insecurity also limited attendance at some of these FJPD offices, thereby somewhat limiting the geographical representation. Third, the findings rest solely on the qualitative sample of 15 and do not generalize beyond institutions similar in nature.

However, the combination of quantitative breadth and qualitative depth offers a nuanced understanding grounded in practitioners’ experiences. This balance between measurement and meaning is likely to increase both the methodological quality of the study as well as its utility for policy (Creswell and Plano Clark, 2018).

### Summary

In brief, this study used an explanatory sequential mixed methods design to examine DV and social policy-making in Palestine. The quantitative phase made statistical links between levels of violence and effectiveness of policies, whereas the qualitative phase shed light on lived experiences and barriers that explain these trends.

Validity of the instruments, consistency in method and ethics, as well as triangulation across methods help keep the findings credible and useful. This method is consistent with social policy and gender research international practice, and thus provides a solid empirically-based tool to press for more homogenous reforms in Palestine and elsewhere.

## Results

### Overview of data and participant characteristics

One hundred and sixty-nine officers from across the West Bank and Gaza Strip working in FJPD were included in this study. Their demographic and occupational diversity of the respondents would also facilitate more equitable inquiry on social policy implementation” “The attitudes toward domestic violence could have been balanced as well since men and women from different kinds of work had participated in this study.

Of all respondents, 61% were male and 39% female, proportions similar to the general gender balance in the department. Participants’ average age was 36.7 years (SD = 7.9, range = 24–55). 52% had a bachelor’s degree, 27% diplomas and 21% master’s degrees or higher. Five to 10 years of professional experience was the average for most (58%); followed by more than 10 years (30%). Seventy per cent were based in the West Bank and 30% in Gaza.

This range of diversity facilitated inferential contrasts, and highlighted the extent to which the sample was representative. Crucially, because we were able to achieve gender and governorates balance, it was possible to consider any potential differences in perceptions—a neglected element of previous Palestinian studies (Rahmeh, 2022).

In addition to statistical diversity, the class composition of participants mirrors institutional realities of the Family and Juvenile Protection Department. The police officers and welfare workers who took part in this research worked across diverse operational contexts, from urban areas with well-established protection teams to rural areas where services are sparse and community mediation is the norm. This difference adds depth to our understanding of how professional background and geographic location influence respondents’ perceptions about DV and policy efficacy.

This contextual information is essential to unpack the results, as it places the figures in the daily professional and personal life people experience while working under different social, political and logistical conditions throughout Palestine. Recent institutional reports also underscore such demographic and regional inequalities in protection services, including the inequal allocation of resources between urban and rural areas (UN Women, 2024a,b; WCLAC (Women’s Centre for Legal Aid and Counselling), 2024).

### Descriptive findings: patterns and perceptions of domestic violence

Analysis of the first domain forms and prevalence of domestic violence-revealed consistently high perception levels across all dimensions. The overall mean score was 4.12 (SD = 0.64) on a 5-point scale, indicating that respondents generally agreed that domestic violence remains a pervasive issue within Palestinian families.

Among the specific dimensions:

- Psychological violence had the highest mean (*M* = 4.36, SD = 0.55), most respondents said that emotional manipulation, insults and verbal humiliation are prevalent. Officers underlined that these practices are commonly accepted by marital relationships, which makes them so hidden and invisible kinds of abuse.
- Economic violence succeeded (*M* = 4.18, SD = 0.61), and numerous officers said that they have witnessed repeated instances when husbands deny women monetary autonomy, refuse them jobs or command over the earnings.
- Physical violence was rated moderately-to-high (*M* = 3.98, SD = 0.72), whereas social isolation (*M* = 3.95, SD = 0.68) was also rated that reflects the continued inhibition of movement and socialization among women.

These results correspond to the national trends that show that psychological and economic abuse is prevalent in the reported cases (Palestinian Central Bureau of Statistics (PCBS), 2024). The high rate of non-physical violence supports previous studies according to which cultural norms tend to legitimize emotional and financial dominance and stigmatize the free admission of physical abuse (Rahmeh, 2022; Dwekat, 2019).

Collectively, the descriptive results confirm that domestic violence in Palestine remains systemic and multifaceted, operating within both overt and covert social mechanisms.

In addition to the numbers, participants often commented on the human price of such types of violence. In describing the cases of officers, the victims had developed longer than anticipated psychological distress and social isolation, but they did not seek help of the law because of the fear of being labeled or feared. Several participants noted that domestic violence, particularly psychological and economic abuse, is often viewed as a private family matter rather than a public concern a perception that continues to silence many women.

Recent institutional reports reinforce these findings, emphasizing that domestic violence in Palestine persists not only because of weak enforcement mechanisms but also because of deeply ingrained cultural acceptance that discourages reporting and formal intervention. Both UN Women (2024a,b) and WCLAC (Women’s Centre for Legal Aid and Counselling) (2024) emphasize that a high percentage of survivors choose reconciliation or informal mediation in order to escape backlash in the community. This fact highlights the fact that prevalence rates involve not only the abuse frequency but also the permanence of the silence around it.

In this context, the descriptive findings move beyond statistical prevalence to capture the lived reality of domestic violence-an everyday struggle shaped by social pressure, institutional constraints, and the normalization of harm within intimate spaces.

### Perceptions of social policy effectiveness

The second analytical dimension examined officers’ evaluations of the effectiveness of social policies addressing family protection and domestic violence. The composite average of this field was 3.61 (SD = 0.70), which showed a moderate level of satisfaction with the existing frameworks of policy.

Respondents generally acknowledged the existence of legislative and institutional structures, such as the Family Protection Units and the proposed Family Protection Law. Nevertheless, implementation capacity and institutional coordination were always rated low by them.

The subscale policy clarity and legal framework got a fairly high score (*M* = 3.95, SD = 0.58) which indicates that most of the participants were cognizant of whether there were related laws and regulations. On the contrary, the least mean (*M* = 3.25, SD = 0.82) was in the category of resource adequacy and infrastructure, which notes that there are serious concerns regarding the lack of adequate budgets, shortages of employees, and restricted access to shelters-the results also correspond to Abu Senina (2020).

Respondents further remarked on how national policy statements and local implementation are constantly out of touch, especially in rural or more conservative communities where legal requirements are frequently compensated by traditions. The subscale on “public awareness and community engagement” yielded a mean score of 3.43 (SD = 0.74), underscoring the limited reach of official outreach programs and the persistence of social stigma surrounding domestic violence.

The written remarks and interviews of the participants also provided an example that the effectiveness of policies is often viewed in the context of daily operational issues rather than legal documents. The officers mentioned sluggish responses as the result of bureaucratic overlap, lack of well-defined referral systems as well as emotional impact of work without sufficient institutional support. They reiterated severally that legislations cannot work unless they are supported with effective coordination and a continued supply of resources.

The professional observations are resonated by the recent reviews of the policy. As noted by UN Women (2024a,b) and WCLAC (Women’s Centre for Legal Aid and Counselling) (2024), the legislative drafting is always large, but enforcement mechanisms are still immature, and the interaction between the government and civil society is usually informal. Both reports stress that the effectiveness of domestic violence policy in Palestine depends less on the existence of frameworks and more on their translation into practice through trained personnel, community trust, and stable funding.

Overall, the quantitative and qualitative evidence together suggest that policy design in Palestine has advanced faster than institutional capacity, resulting in uneven implementation across regions a trend observed in other post-conflict and resource-constrained contexts (UNFPA, 2022; Sanioura, 2022). The disorienting distance between policy aspiration and actual experience still occurs as a factor in influencing officers to develop tentative assurance: on the one hand, they acknowledge improvement in theory but on the other hand, they are skeptical about its effectiveness in practice.

### Inferential statistics group differences

The independent-samples t-tests and one-way ANOVA, were used to determine the variation in perceptions across demographic groups.

### Gender-based differences

Results revealed no statistically significant difference between male and female officers regarding their perceptions of domestic violence prevalence (*t*(167) = 1.42, *p* > 0.05). But the female officers expressed concern related to institutional barriers a little bit more (*M* = 4.22) than the male officers (*M* = 4.01).

This pattern echoes previous findings that women professionals, particularly those working in gender-related fields, tend to show greater sensitivity to the systemic nature of domestic violence and advocate more strongly for policy reform (Rahmeh, 2022; UN Women, 2023).

Emotional fatigue and frustration based on the discrepancy between professional ideals and institutional constraints was also reported by female officers in interviews. They highlighted that gender dynamics within the police hierarchy sometimes restrict their influence in decision-making, especially on cases involving women’s protection. These perspectives underline that gender differences, while not statistically large, carry qualitative weight in shaping how officers interpret and respond to domestic violence.

### Level of education and experience

The analysis of variance (ANOVA) showed that there existed significant differences based on education (*F*(2,166) = 4.23, *p* < 0.05). Master degree holders reported more dissatisfaction with policy performance than their wizard degree counterparts did, implying that the more academically exposed one is, the more critical of the institutional system (Rahmeh, 2022; UNFPA, 2022).

No significant difference was also achieved in professional experience (*F*(2,166) = 3.58, *p* < 0.05). The higher the levels of policy stagnation among officers with 10+ years of experience, probably due to frustrations of being long-term exposed to the restrictions of bureaucracy.

These results indicate that education and experience are not only related to technical knowledge, but also to the emotional and analytical perspective of policy that practitioners can use. The members of the senior officers, formed over years of difficulties in the field and the failure to bring change, frequently spoke of a feeling of policy fatigue, whereas more junior, or inexperienced officers, spoke more of a feeling of gradual institutional improvement. This generational difference indicates the impact of socialization in the field of profession on change expectations.

Equally the higher educated officers stated that the international exposure to international structures and human rights rhetoric made them more conscious about policy loopholes as they grew more outspoken on policy change. UN Women (2024a,b) affirms that, important as it is, this kind of professional awareness may become frustrating when institutional processes are not responsive or supported by political parties.

Combined with the effect of educational level, the effect of experience in years underlines the fact that knowledge in itself does not bring reform. It is important also to note how well the institutional environment is prepared to put expertise into a meaningful policy action.

### Regional variations

A t-test of West bank and Gaza respondents showed that there were significant regional variations within the region (*t*(167) = 2.74, *p* < 0.01). Gaza officers expressed less satisfaction (*M* = 3.28) than West Bank officers (*M* = 3.69). This gap can be explained by political fragmentation, economic hardship, and dissimilar structures of authorities, which were described in previous research (Sanioura, 2022; UNFPA, 2015).

Respondents in Gaza explained that they were forced to perform their duties in harsh material and psychological conditions, such as scarce resources, high caseloads, and weak connections with civil bodies. It was widely observed that the implementation of policies is often nominal as opposed to operational because, in the day-to-day survival, institutional planning is pushed aside in favor of daily survival. West Bank officers on the other hand enjoyed slightly better NGO associations and training sessions that improved coordination despite the same cultural constraints.

These differences are affirmed in recent policy assessments by UN Women (2024a,b) and WCLAC (Women’s Centre for Legal Aid and Counselling) (2024) that underscore the fact that political division and unequal donor financing to Palestinian areas have established unequal systems of protection. In Gaza, officers are often employed without a stable referral system or access to shelter, whereas, in the West Bank, they are institutionally better supported but deal with a sociocultural opposition.

The results of this research show that regional difference is not just a statistical fact but an experiential reality which informs the way protection policy is read and practiced. Successful reform thus needs to be region sensitive-based on resources, training and models of coordination to local issues of each administrative setting.

In sum, demographic and regional variations underscore the contextual complexity of domestic violence policy implementation, reinforcing the need for localized and adaptive approaches rather than uniform national models.

### Correlation and predictive analysis

To determine the relationship between domestic violence prevalence and social policy performance, Pearson’s correlation coefficient (*r*) and multiple regression analysis were applied.

The results revealed a strong negative correlation between perceived domestic violence prevalence and policy effectiveness (*r* = −0.61, *p* < 0.001). In other words, the higher the perceived levels of domestic violence, the lower the perceived effectiveness of social policies addressing it. The finding confirms the premise behind the hypothesis on the inverse relationship and aligns with the evidence provided in other post-conflict societies in other countries (Haider, 2018).

This negative correlation reflects a cycle where institutional weakness and gender-based violence reinforce one another. It was also noted that when the victims lack trust in the protection systems, they fail to report the cases and reduce the institutional visibility and further worsen the response to policies. Absence of institutional presence, in its turn, allows violence to remain wild, and lowers the levels of trust of people in social protection systems. Such a reciprocity is desirable of a weak governance environment as suggested by UN Women (2024a,b) and WCLAC (Women’s Centre for Legal Aid and Counselling) (2024).

The policy effectiveness (dependent variable) was then predicted by four independent predictors by multiple regression analysis:

- Psychological violence
- Economic violence
- Physical violence
- Institutional barriers

The regression model was significant (*F*(4,164) = 18.93, *p* < 0.001), explaining 43% of the variance (Adjusted *R*^2^ = 0.43). Among predictors, institutional barriers (*β* = −0.47, *p* < 0.001) and psychological violence (*β* = −0.31, *p* < 0.01) emerged as the strongest negative influences on policy performance.

This will mean that institutional barriers such as lack of resources, lack of coordination and training will only weaken the confidence between the social policies. Foretelling quality of psychological violence reflects its widespread and culture-affirmative nature that poses more challenges to the organization of formal institutions by conventional means.

Participants’ qualitative reflections confirmed these quantitative results: officers repeatedly emphasized that institutional barriers especially the absence of a ratified Family Protection Law create confusion in case management and weaken the authority of field officers. On the same note, the normalized character of psychological abuse presupposes that the majority of the victims do not address such behavior as the different manifestation of violence, thus it becomes even harder to prevent and eradicate.

These intertwined factors demonstrate that domestic violence is both a cause and a symptom of weak governance. The answer to it then must be not only improved legislation but also more cultural change, interagency coordination, and institutional accountability.

The results therefore support the study’s primary hypothesis: domestic violence significantly affects the formation and implementation of social policies in Palestine, particularly through the mediating influence of institutional fragility and sociocultural normalization.

### Qualitative phase findings

The qualitative stage added some contextual relevance to the statistical outcomes. The thematic analysis of 15 interviews was able to find three large themes that matched the barriers and dynamics that were found in the quantitative data. The participants expressed the emotional and institutional realities which stand behind the numbers through these narratives, the daily conflict between professional responsibility and cultural norms.

### Theme 1: violence as a culture

The respondents outlined general social approval of patriarchal rule in which physical chastisement and emotional repression are viewed as fine manifestations of discipline. According to the officers, victims tend to retract complaints under family pressure or social ostracism. This acceptance not only hinders legal enforcement but leaves the prevention work to no effect, because the elders of the community or religious leaders are able to resolve the cases outside the protection agencies (Dwekat, 2019; Rahmeh, 2022).

There were repeated observations that law is always second to custom particularly in small and traditional towns. The social reputation and family honor impacts keep the women not to report any form of abuse and informal reconciliation mechanisms would be more focused with maintaining social stability rather than on personal safety. WCLAC (Women’s Centre for Legal Aid and Counselling) (2024) and UN Women (2024a,b) have equally reported that the existence of these informal settlements has continued to reinforce the cycle of silence and fear that constrains institutional intervention and undermines trust in its official processes.

### Theme 2: institutional resource constraints

The shortage of human and financial resources, seen as chronic by all the participants. Most offices do not have specialized social workers or psychologists and the police officers are left to play outside their comfort zone. The institutional coordination, particularly between FJPD, ministry of social development and non-governmental organizations was reported to be fragmented and inconsistent. Another reason mentioned by officers was a lack of standardized databases in which officers could track cases, resulting in duplication or data loss. These results support quantitative data that resource insufficiency is a leading disadvantage to policy effectiveness (Abu Senina, 2020; UNFPA, 2015).

The participants noted that these shortcomings are not only logistical, but also moral. Due to the absence of infrastructure and case management systems, frontline officers develop a feeling of helplessness. According to a respondent, we as a people desire to be safeguarded, however, in some cases, the system fails to safeguard us. This observation is consistent with Palestinian Working Women Society for Development (PWWSD) (2021), as well as UN Women (2024a,b), which underscores that despite the best laws, which are progressive but not practical without resources and coordination.

### Theme 3: emotional burden and burnout

The professionals found that the complainants were the individuals who were emotionally fatigued due to repetitive traumatic stories, absence of any psychosocial support, and personality assault within the community. Other officers also reported intra-operational conflicts based on which they perceived their professional jobs to be incompatible with societal norms and causing them moral discomfort. This is a negative impact on motivation, and long-term implications of policy sustainability (Rahmeh, 2022; UNFPA, 2022).

Some of the officers reported being affected in emotional terms of the job stating that they had insomnia, compassion fatigue and even moral fatigue. This is contributed by the lack of institutional counseling or peer-support programs. These testimonies illustrate that protection work in Palestine is not merely administrative but deeply human an act of endurance within a system still struggling for coherence and empathy (WCLAC (Women’s Centre for Legal Aid and Counselling), 2024; UN Women, 2024a,b).

Collectively, the qualitative findings illuminate the human dimension behind statistical trends demonstrating how cultural, structural, and emotional factors intersect to shape the lived reality of domestic violence intervention. They remind the policymakers that the reform is not only a legislative and resource issue but an issue that accompanies the people to whom it turns out as an emotional baggage to safeguard other fellow human beings.

### Integration and interpretation of quantitative and qualitative findings

Integrating both data strands reveals a coherent yet complex picture:

- Quantitative results established the magnitude of domestic violence and its inverse relationship with policy effectiveness.
- Qualitative insights contextualized these patterns by uncovering the cultural and institutional mechanisms sustaining this relationship.

The findings confirm that domestic violence in Palestine is both a social symptom and a structural condition. Although quantitative information was used to measure the prevalence and policy disparities, qualitative stories illustrated the role that permissive patriarchal norms and institutional frailty play in keeping the cycle alive. All these findings make it clear that figures cannot reflect the entire complexity of the problem, of course, each statistic is a live narrative of constraint, adaptation, negotiation on the edges of a weak framework.

Three integrative observations emerge:

1. Policy-Practice Gap: This is the gap between a stated policy existing and a deficiency of ability to implement it, which is the symptom of the so-called symbolic compliance pattern—whereby organizations declare their progressive frameworks but lack the capacity to implement them (UN Women, 2024a,b; Haider, 2018). During the interviews, this was also called a paper policy problem; the interviewees cited that there is statute, and departments are documented on paper but there is the absence of the authority and resources to execute the initiated strategies. As UN Women (2024a,b) and WCLAC (Women’s Centre for Legal Aid and Counselling) (2024) confirm, the tendency is granted across the region, and the institutions do not pay attention to the procedural changes but formal adherence.
2. Contextual Determinants: Regional and political conditions especially in Gaza compound gender-based violence through economic insecurity and fragmented governance (Sanioura, 2022). Occupation, political division and patriarchy culture become amalgamated together creating a complex environment where patients and practitioners are constantly confronted with uncertain circumstances. At Gaza, officials claimed to operate without a system and at the West Bank they claimed to operate in a system which does not necessarily work. The duality defines the direct influence of the protection capacity of political geography (Palestinian Working Women Society for Development (PWWSD), 2021; UN Women, 2024a,b).
3. Human Resource limitations: Human jadedness in the protection officers undermines the sustainability of the policy meaning that wellbeing of the staff is an extremely crucial but neglected element that determines policy success (Abu Senina, 2020). This aspect of human factor that is never addressed in policy documents was a serious determinant of effectiveness. Practitioners become the vulnerable place in the protection systems as they are exhausted, unsupported, or stigmatized. Caregiver institutional care is therefore not a luxury but a prerequisite to a sustainable reformation (WCLAC (Women’s Centre for Legal Aid and Counselling), 2024).

Combined conclusions indicate that the sustainable development involves the need to take action, at a variety of levels:

- Formal changes to block loopholes in the law and standardize implementation strategies.
- Raising institutional capacity to build coordination, monitoring and accountability.
- Cultural education Community education that challenges cultural normalization of violence and empower survivors.
- Psychosocial support of the workers and their psychological integrity and resilience to defend other people.

Ultimately, the convergence of quantitative and qualitative evidence confirms that the challenge of domestic violence in Palestine is not solely a matter of law or policy but of human systems, trust, and the moral will to translate written frameworks into lived protection.

### Summary of findings

The analysis is providing four main empirical findings:

1. Domestic violence is widespread and multifaceted, with psychological and economic abuse being the most prevalent forms. These forms of violence often go unnoticed because they are embedded in everyday social interactions and justified through cultural norms, reflecting the deep normalization of gendered control within Palestinian society.
2. Effectiveness of the perceived policy is moderate, and the main disadvantage of its implementation is poor coordination and insufficient resources. Although policy and protection units are formally established, their areas of operation are only limited by lack of coordination and the lack of funding resources that are reflected by national evaluation by UN Women (2024a,b) and WCLAC (Women’s Centre for Legal Aid and Counselling) (2024).
3. The most powerful negative predictors to policy success are institutional barriers and psychological violence. It implies that the less confidence officers place in institutional coherence and the less naturalized the psychological violence is, the less likely it is that the perception of policies producing actual change exists.
4. Qualitative results reflect the mutuality of cultural normalization, institutional weakness and practitioner burnout as the determining variables of policy outcomes. All these human and structural forces emphasize the fact that proper protection cannot be achieved only with legal frameworks but with the strong and emotionally supported staff.

Collectively, these results confirm the central thesis of the study: domestic violence in Palestine directly shapes and is shaped by the processes of social policy formation and implementation. It works both as a sign of larger inequality and an obstacle to change.

Therefore, real policy improvement cannot be based on legislation sexually but a multidimensional change that fills the divide that exists between legal structures, institutional practice and belief systems in the society. It is necessary to reinforce cross-sectoral coordination, empowerment of practitioners and community awareness in order to transform the intent of policy into protection reality (Palestinian Working Women Society for Development (PWWSD), 2021; UN Women, 2024a,b).

## Discussion

### Overview

The findings of this study reveal a complex and often uneasy relationship between domestic violence and the formation and implementation of social policies in Palestine. Domestic violence emerges here not simply as a pattern of behavior within individual households but as a symptom of deeper structural inequalities and institutional fragilities that shape everyday life. Combining both the quantitative and qualitative evidence, the paper shows the intersection of the violence prevalence, the policy structure limitations, and the actual life experiences of numerous practitioners in the occupation, politically divided world and the patriarchal commanding forces that have their roots in the deep-rooted patriarchal environment.

Overall, the mixed-methods design confirmed a strong negative relationship between the perceived prevalence of domestic violence and the perceived effectiveness of social protection policies. This pattern is consistent with research in other post-conflict and patriarchal settings, where social policies often struggle to address gendered violence under conditions of scarce resources, weak institutions, and resistant social norms (Haider, 2018; Sanioura, 2022). Recent Palestinian and international assessments echo this tension, noting that despite policy commitments, domestic violence remains widespread and under-reported (Palestinian Central Bureau of Statistics (PCBS), 2019, 2024; UN Women, 2024a,b; WCLAC (Women’s Centre for Legal Aid and Counselling), 2024).

### Interpreting the prevalence and nature of domestic violence

Psychological and economic violence are at the highest (*M* = 4.36 and *M* = 4.18 respectively) suggesting that non-physical violence is mainly normalized in Palestinian families. These results support the previous literature that asserted that emotional and financial control are some of the most important means by which patriarchy is enacted and preserved, especially in cultures where masculinity is linked with authority, providence, and discipline (Dwekat, 2019; Rahmeh, 2022; UN Women, 2024a,b).

Psychological abuse humiliation, threats, verbal intimidation, and systematic belittling emerge as the “invisible backbone” of domestic violence. Since these practices are usually repositioned as a male of marital strife or as an authorized chastisement, they are more difficult to identify and to contest, both within society and within the institutions. This is one of the reasons why formal responses are usually reactive, emphasizing dramatic physical events over the daily dissolution of dignity and autonomy. This trend is strengthened by economic violence: limiting access to income, blocking employment opportunities, and decisions about finances all contribute to further dependency of women and their inability to leave abusive relationships (Rahmeh, 2022; UNFPA, 2022).

These dynamics are consistent with feminist theory, which understands domestic violence as a mechanism for preserving gender hierarchy rather than an isolated behavioral problem (Dobash and Dobash, 1992). These hierarchies are not only perpetuated by the power of men in Palestinian families, but also by high demands that women keep their families united and honorable, even by sacrificing their own security level. Violence is commonly accepted as a remediating process in this moral economy, and silence is accepted as a sign of loyalty and righteousness (Palestinian Working Women Society for Development (PWWSD), 2021).

According to the social learning perspective (Bandura, 1977), intergenerational cycles are also indicated in the outcomes. Policemen reported numerous instances of offenders in which designers had been raised in a violent family and seemed to have internalized violence as a natural or effective method of resolving conflict. This is consistent with regional research linking childhood exposure to domestic violence with higher acceptance and perpetration in adulthood (Veronese et al., 2025; Salameh, 2025).

Taken together, the persistence of domestic violence in Palestine reflects an intersection of psychological, cultural, and structural determinants. Any effort to counter it using punitive interventions will be thus an inadequate response. Legal reform needs to be supported by educational, economic and cultural interventions that provoke the meaning to the authority, obedience and “protection” in family life.

### Policy effectiveness and institutional capacity

The moderate value of the policy effectiveness (*M* = 3.61) indicates the obvious distance between the design of the policy and the reality of its operation. Participants acknowledged the existence of legislative and institutional frameworks such as Family Protection Units and the long-debated Family Protection Law yet repeatedly pointed to weak implementation, limited authority, and fragmented coordination. This trend constitutes a conceptualized policy-practice gap whereby governments espouse progressive policies, at times under international compulsion, without the political support, investment or facilities to effect any pertinent action (Merry, 2006; UN Women, 2024a,b).

The lowest overall score in the domain, concerned with the areas of resource adequacy and infrastructure (*M* = 3.25), addresses the continued lack of funds, specialist workers, and secure facilities. These constraints mirror broader regional trends in which family protection units operate with minimal budgets and rely heavily on donor-supported projects rather than stable public finance (UNFPA, 2015; Ministry of Social Development, 2023).

Within this landscape, the absence of an enacted Family Protection Law stands out as a structural barrier. The draft law is still not adopted after almost 20 years, and it creates a legislative gap that compromises the accountability of institutions and eroding the power of frontline officers. Consequently, reaction to violence has been seen to rely on discretion of judgment as opposed to legal compulsory approaches especially in either country (rural or conservative) where traditional reconciliation and familial mediation prevail over legal provisions. The recent advocacy reporting by WCLAC (Women’s Centre for Legal Aid and Counselling) (2024) emphasizes that fragmented policies and uneven procedures are the direct results of the absence of a single legal reference, whereas UN Women (2024a,b) mentions that currently, the interventions of domestic-violence are strongly reliant on donor funds and non-binding administrative coordination.

Such structural weaknesses are also intensified by the fact that the problem of patriarchal legacy of political and bureaucratic hierarchies remains. Decision-making circles often prioritize social harmony and political stability over confronting violence, and gender equality is frequently framed as a secondary or externally driven agenda. Palestinian Working Women Society for Development (PWWSD) (2021) records the loss of legitimacy around the protection agencies by the sheer societal acceptance of violence, righteous through the discourse of family honor and family cohesion. In practice, gender-equality commitments risk remaining rhetorical rather than operational, constrained by both sociocultural resistance and legislative inertia.

Meanwhile, the absence of effective organization between the FJPD and the Ministry of Social Development with the Ministry of Women Affairs, and civil-society organizations can also be seen as a larger indication of institutional fragmentation which is reinforced by the political separation between the West Bank and Gaza. There was communication of overlapping mandates, incompatible procedures and lack of shared case tracking systems by the officers- which analysts in line with the study by Abu Senina (2020) have found exhibits duplication and inefficiency due to the absence of unified protocols.

These results point to a deeper cultural-institutional dissonance: while official discourse increasingly invokes gender equality and human rights, everyday practices remain constrained by patriarchal assumptions and concerns about reputation, social cohesion and “family privacy.” As Rahmeh (2022) points out, policies can only be effective in the context of a culture that promotes them. Without engaging the cultural underpinnings of domestic violence, institutional measures risk remaining superficial.

### Understanding intergroup variation

The absence of statistically significant gender differences in perceptions of domestic-violence prevalence (*p* > 0.05) challenges a simplistic assumption that female professionals are inherently more sensitive to gender issues than their male colleagues. The two recognized the systemic nature of violence. Nonetheless, the qualitative data indicate that female officers were more likely to report more empathy with survivors and a greater perception of frustration toward bureaucratic stalemate. This nuance aligns with research indicating that professional role identity, training and lived experience intersect with gender in shaping attitudes toward policy, rather than gender alone determining sensitivity (Haider, 2018; Rahmeh, 2022).

The education and experience difference was amplified. Highly educated officers and those who had a 10-year tenure rated higher on dissatisfaction of policy effectiveness. This trend can be discussed in accordance with the claim that increased academic exposure and previous field experience are likely to increase the critical perception of the institutional strengths and weaknesses (UNFPA, 2022; Rahmeh, 2022). As they had seen a cycle of plans and strategies multiple times, and the outcomes were minimal and not physically measurable, experienced officers spoke of having ‘policy fatigue’. These feelings have been documented in research of practitioners working in structurally constricted settings in Jordan and Lebanon (ESCWA, 2025; UN Women, 2024a,b).

Also, differences in the regions of the West Bank and Gaza depict the influence of political and economic context on the social-policy outcomes. Gaza officers showed a low level of satisfaction (*M* = 3.28) compared to the West Bank (*M* = 3.69), indicating the infrastructural collapse, political isolation and constant crises as key challenges. This result can be echoed with the concept of contextual policy fragility, in which the external political factors and humanitarian pressures prevent the local institutions to administer justice and protection (Sanioura, 2022; UN Women, 2024a,b; WCLAC (Women’s Centre for Legal Aid and Counselling), 2024).

### Linking domestic violence and social policy: a structural perspective

The strong negative correlation between perceived domestic-violence prevalence and policy effectiveness (*r* = −0.61, *p* < 0.001), together with the regression model (Adjusted *R*^2^ = 0.43), provides quantitative support for a central premise of this study: that the prevalence of domestic violence undermines the performance and credibility of social policies. From a conflict-theory perspective, domestic violence mirrors broader patterns of unequal access to power, resources and recognition (Haider, 2018). Once the normalization of violence in the family is reproduced, these dynamics of domination and resignation are replicated between citizens and weak institutions.

The regression outcomes reveal that institutional barriers (*β* = −0.47, *p* < 0.001) were the most negative predictor of policy performance, then there was psychological violence (*β* = −0.31, *p* < 0.01). This implies that the problem of policy failure is not only a factor of social attitudes but is very much rooted in the ability, integrity and responsiveness of the institutional system. The confidence of practitioners in the system to safeguard survivors consequently diminishes where they perceive high levels of bureaucratic obstruction, poor coordination, and lack of training.

Less visible and more widespread forms of abuse, psychological violence also has great policy implications. It is more difficult to detect; it is less easily documented and prosecuted and therefore does not fit traditional monitoring systems. This leads to the deficits of psychosocial services and the discrimination of mental-health aspects on domestic-violence policy (Rahmeh, 2022; Palestinian Central Bureau of Statistics (PCBS), 2024; UN Women, 2024a,b).

In this sense, domestic violence appears both as a cause and as a consequence of weak governance. Normalized violence undermines the perceived credibility of institutions, infringing the willingness to report and reinforcing the utilization of less formalized systems which consequently happens to limit the approachability of formal policies further into the future.

### Qualitative knowledge: cultural and emotional

The qualitative findings complement the statistical depiction in anticipating the cultural and emotional realities in which practitioners operate. The participants repeatedly mentioned social rules, according to which a survivor is not allowed to report violence, and family privacy and reputation are more valuable than their safety. This reflects Shaheen et al. (2020) observation that common sayings and narratives in Arab societies often reinforce gender hierarchy and portray a woman’s endurance as an essential component of honor.

The officers also cited informal mediation by the family elders, tribal leaders and religious figures. Those interventions would reduce short-term tensions, but are more concerned with reconciliation than accountability, and in most instances are inappropriate to the structural conditions underlying the violence. The recent findings of UN Women (2024a,b) and WCLAC (Women’s Centre for Legal Aid and Counselling) (2024) make analogous conclusions, documenting how informal settlements contribute to continues cycles of responsive harm and silence rather than prevent further abuse.

The answers are further complicated by institutional limits—most notably lack of highly-specialized workers and systematic training. The complaints that most officers raised were that, they are ill equipped to handle complex psychosocial cases in situations where such cases involved children or the grossly traumatized survivors. The police officers are likely to be involved in a role that they are not equipped to fulfill with regard to their professional competence, which can only increase the strain on them, also limiting the intervention. Such experiences do not contradict the results of past reviews by UNFPA (2015) and Ministry of Social Development (2023) that multidisciplinary teams and continued capacity building are required.

A particularly bright theme is emotional burden and burnout. The exposure to the traumatic cases over time, and the absence of institutional support, along with the backlash in the community every now and then is what some of the interviewees referred to as the feeling of moral exhaustion. Officers complained of insomnia, a lack of belief in their capabilities of doing anything, and their growing sense that they are no longer demonstrating any attachment to their professional ideals and what the system will allow them to achieve. This scenario can be considered within the context of the Job needs Demands-Resources in that it involves a combination of high emotional surges and a lack of organizational assistance that reduces interference and performance with time (Bakker and Demerouti, 2007).

These qualitative insights underscore that policy implementation is not only a technical or administrative process. It is also emotional labor and it is dragged by practitioners who on their side are exposed to the pressures and both ends of a sensitive mechanism. Their wellbeing should then be put into consideration by any such institutional reform.

### Integrating theoretical perspectives

These inferences are based on, as well as generalized by the research paradigms used in directing the research. Feminist theory helps explain the persistence of domestic violence by pointing to patriarchal structures embedded in both family life and state institutions. Findings of the studies have shown that such structures not only justify abuse, but also predetermine the institutional reactions, such as focusing on reconciliation instead of accountability or protection as a discretion being made instead of a right (Dobash and Dobash, 1992; True and Hewitt, 2022; Rahmeh, 2022).

The social learning theory explains the transference of the violent behaviors to generations. The fact that, the number of perpetrators was likely to have violent childhood as officers perceived it, creates the image of how the pattern of aggression and dominance are offered to children that will eventually become the seed that form their relations (Bandura, 1977; UNFPA, 2022). Such processes are seen through the set of the conflict theory circumstances in the bigger picture of power, resource, and legitimacy competition within the occupationally and politically polarized setting, in which women are vulnerable to structural and intimate violence (Haider, 2018; Sanioura, 2022).

Taken together, these frameworks suggest that domestic violence in Palestine is best understood as a structural condition rather than an individual pathology. It coincides and advances imbalanced power relations, and, in the process, suppresses the possibility of reforming social-policy.

### Making comparison with local and international literature

The study’s results are broadly consistent with regional and global literature on domestic violence and social policy, while also highlighting specific features of the Palestinian context. Compared to the data on the world according to World Health Organization (2021), which is one woman in three in cases of experiencing physical or sexual intimate partner violence, Palestinian data reveal the high rate of psychological and economic abuse (Palestinian Central Bureau of Statistics (PCBS), 2019, 2024). This deficiency underscores temporary impacts of political unsteadiness, economic uneasiness and male rigidity on the existence of ladies (Sanioura, 2022; Haider, 2018).

Similar to evidence from Jordan and Egypt (ESCWA, 2025; Rahmeh, 2022), this study shows that domestic violence is not a purely private issue but a matter of public concern and policy design. However, unlike relatively more stable neighboring states, Palestine’s institutional capacity is constrained by occupation, internal political division and heavy donor dependence. Therefore, the social-policy practices are inclined to balance between the symbolic reform that is meant to demonstrate the compliance with the international standards, and the emergency responses.

The outcomes worldwide are consistent with the absence of implementation discourses among the low- and middle-income mothers that the well-motivated legislation has failed to yield any fruits since the implementation machineries are inefficient, the scattered sectors and limited budgets (Merry, 2006; UN Women, 2024a,b). The Palestinian case, however, adds the dimension of “conflict-induced gender inequality,” where domestic violence is simultaneously a product of gender hierarchy and a by-product of chronic geopolitical instability (Haider, 2018; True and Hewitt, 2022).

### Policy practice relations: symbolism to practical reform

The results highlight a policy environment marked by what can be called symbolic compliance: institutions adopt progressive frameworks such as the draft Family Protection Law and national strategies on gender equality yet their implementation remains partial and uneven (UN Women, 2024a,b; WCLAC (Women’s Centre for Legal Aid and Counselling), 2024). It is the physical manifestation of this notion and how officers perceive poor coordination, powerlessness, and resources.

Both the social mentality and institutional mechanisms can be observed in the shift of the symbolic to substantive reform. At institutional level, distinct roles split and formalized coordination of the FJPD, the ministry of the social development, ministry of the women affairs and civil-society organizations are essential. Shared digital databases and unified referral routes would minimize the duplication; accountability would increase (UNFPA, 2015; Ministry of Social Development, 2023).

Socially, the society should be engaged to change the debate about victim-blaming and family honor to the concept of rights, protection and shared responsibility. Community-based awareness programs, involving religious leaders, educators and local councils, can help reframe domestic violence as a violation of human dignity rather than a private dispute (Palestinian Working Women Society for Development (PWWSD), 2021; UN Women, 2024a,b; Rahmeh, 2022).

Within this broader process, the ratification of the Family Protection Law remains a pivotal step. In line with recent national and UN assessments (UN Women, 2024a,b; WCLAC (Women’s Centre for Legal Aid and Counselling), 2024), the present study underscores that the law is not simply a legal instrument but a foundation for coherent, enforceable and sustainable policy implementation. Its adoption however, with some discussion publicly, education of the professions of with a system of mechanisms of monitoring and enforcement, is to be effective. The law should be reformed along with a social change: law can provide structure and power but culture change will provide legitimacy and permanence.

### Institutional and human dimensions of policy implementation

Among the findings of the study that are the most relevant is related to the emotional labor of the practitioners. The tensions that are experienced by officers who work in the FJPD on daily basis, are between professional standards, institutional restraints and social expectation. There were many cases stated when I was aware of what should have been done in regard to protection parameters and could not since I had no resources, did not find out how to do it or was pressured by families and leaders of the community. This ethical distress has even been reported to be a source of frustration, burnout and withdrawal in case of chronic (Bakker and Demerouti, 2007; UNFPA, 2022; Rahmeh, 2022).

This suffering is further enhanced in the greater political-economic context of Palestinian setting. Gaza practitioners, particularly, are busy with big numbers of cases, poverty and the occasional blockage of services. In the meantime, in both the police are at a risk of societal revenge when they intervene in family matters, which are considered sensitive. These strains emphasize the importance of considering practitioners as policy producers and persons whose health are the pillars to effective policy.

They can also consider establishing confidential counselling services, peer support groups and regular debriefing in the FJPD and other partner institutions as a way of promoting their wellbeing. As seen elsewhere across the world, this type of activity helps to keep motivation levels high, reducing the turnover and increasing interaction with survivors (World Health Organization, 2021; ESCWA, 2025). Increasing access of more women to protection units and higher decision-making roles can also be helpful to increase the confidence of survivors and make policy making more diverse (UN Women, 2024a,b; WCLAC (Women’s Centre for Legal Aid and Counselling), 2024).

### Synthesizing the quantitative and qualitative knowledge: to a multilevel model

By bringing together statistical analysis and narrative accounts, this study proposes a tentative multilevel understanding of the domestic-violence-policy nexus in Palestine.

At the micro level, domestic violence manifests in the dynamics of intimate and family relationships psychological harm, economic dependency, and learned patterns of control.

Institutional weaknesses at the meso level (including a lack of enough resources, disjointed coordination, and burnout) influence the manner and degree of policy implementation.

Macro level is the obstacles of what is politically and administratively possible and is influenced by forces of structure, i.e., patriarchy, political instability, occupation and donor dependence.

These levels are not independent of each other. They are found in feedback mechanisms: social norms, which devalue violence, reduce pressure on institutions to act; institutional failure, in turn, supports the notion that the population is skeptical and will not report, as well as policy stagnation, in turn, allows supporting the notion that the violence is something that cannot be changed. The quantitative evidence of this cycle (*r* = −0.61; *β* of institutional barriers = −0.47) supports the empirical evidence of this cycle and the qualitative descriptions of this cycle as experienced and negotiation in practice.

### Contributions to knowledge

There are several contributions to practice and research in this paper. It lacks empirical emphasis and this has rendered it one of the few Palestinian studies that simultaneously draw on quantitative and qualitative information founded on experience of practitioners in the heart of the protection apparatus. The view paper is a bridge between the literature on legal and policies and practice on the ground.

Theoretically, the study refines feminist and conflict-oriented analyses of domestic violence by showing how gendered violence and weak governance mutually reinforce one another in a conflict-affected context. It suggests an integrated framework that may be relevant to other societies experiencing both political instability and deep-seated gender inequality.

From a policy standpoint, the findings provide context-specific evidence that can inform national debates on the Family Protection Law and on the design of gender-responsive social protection strategies. The methodology that shows the advantage of statistical inclination of lived experience to understand the intricate social reality in vulnerable situations is the explanatory sequential mixed-method design.

### Limitations of the study

Despite the strengths, the study has many limitations. One, self-reported questionnaires are able to compromise social desirability bias especially in the face of domestic-violence and institutional-performance questions that make the respondents to be sensitive (Tourangeau and Yan, 2007). Second, the cross-sectional research design only quantifies perception at a single time date without causal evaluation. Longitudinal research would be a better study to observe the transform in the policy and practice.

Third, the qualitative sample was sufficient since it was thematically saturated, but included 15 participants, which is limiting on the perspectives. Fourth, political and logistical constraints limited access to some areas as well, particularly in some geographic locations of Gaza, potentially affecting the geographic distribution of the sample. Finally, the study is about the perception of the practitioners rather than experience of survivors themselves; a perspective of the survivors incorporated in the future practice would provide a more critical assessment of how the system works and fails.

By acknowledging these shortcomings, the study becomes easier to access, and the area where the study should be interpreted becomes clear.

### Implications to policy and practice

The findings have several practical implications to the policy makers, institutions and the international partners.

First, there is an urgent need to ratify and implement the Family Protection Law. The law should provide a comprehensive and gender-sensitive definition of domestic violence, establish clear procedures for prevention, protection and prosecution, and guarantee survivors’ access to support services. Detailed and specific regulations should also provide them to make it a work plan, rather than a performance on paper (UN Women, 2024a,b; WCLAC (Women’s Centre for Legal Aid and Counselling), 2024).

Second, there should be strengthened institutional coordination. A permanent national coordination mechanism on domestic violence bringing together the FJPD, the Ministry of Social Development, the Ministry of Women’s Affairs and key civil-society actors could harmonize referral systems, case-management tools and data platforms.

Third, frontline officers must be skilled in trauma-informed practice, legally literate and engaged in the community just as any other officer would be in need of capacity-building. The training should not be project-based and should be on-going, as well as should consider the special challenges of working in conflict-prone environments.

Fourth, it ought to take into consideration community engagement campaigns against the normalization of violence and stigmatization of the survivors using cultural narratives. Collaboration with religious authorities, educators, local councils and women’s organizations can help shift understandings of domestic violence from a private issue to a matter of public responsibility and rights (Rahmeh, 2022; Palestinian Working Women Society for Development (PWWSD), 2021; UN Women, 2024a,b).

Fifth, psychological practitioner support should be involved in institutional planning. Internal counselling services, peer-support groups and supervision structures can help mitigate burnout and maintain motivation, thereby improving both staff wellbeing and policy implementation (Bakker and Demerouti, 2007; UNFPA, 2022; ESCWA, 2025).

Finally, it is recommended that donors should invest in non-dependent institutional development by initiating long-term projects, data and staff capacity building, and others and support ownership over dependency (UNFPA, 2015; Ministry of Social Development, 2023).

### Future research directions

Whether it is the further research, there are the number of opportunities left to the analysis. Future research could adopt longitudinal designs to evaluate the impact of new laws or institutional reforms on patterns of domestic violence and on trust in protection actors. In addition to that, more is needed regarding victim-centered research and survivor-led research that would study the response or lack of responses to the needs, fears, and expectations of the individuals who are the most affected by the institution in question.

Comparative analysis against other Arab countries may demonstrate similarity of problems and set of promising practices and research of digital tools and social media could help study their role in raising awareness, reporting and mobilization of communities. Further researches on organizational wellbeing, in practitioners, moral distress and resilience, would provide valuable details on the way to strengthen human pillars of protection systems.

By pursuing these directions, scholars and policymakers can continue to build a more nuanced and robust evidence base for gender-responsive policy development in Palestine and in other fragile and transitional societies.

## Conclusion

This research aimed to explore how domestic violence shapes and is shaped by social policy in Palestine, as mediated over gender, governance and institutional reform. It used an explanatory sequential mixed-method design which gathered quantitative data from 169 Family and Juvenile Protection Department (FJPD) officers to inform qualitative interviews with 15 practitioners in order to place statistical patterns alongside the lived experience. Collectively, these views demonstrate that domestic violence in Palestine is not simply a private or interpersonal issue but rather a structural problem which has been exacerbated by political division, economic insecurity and entrenched patriarchal values.

The results consistently show that high domestic violence is associated with lower perceptions of policy effectiveness. Correlational analysis the correlation between domestic violence prevalence and perception of the performance of social protection policies was very strong and inverse (*r* = −0.61, *p* < 0.001), and regression models found that institutional barrier (*β* = −0.47) and psychological violence (*β* = −0.31) were the most salient predictors of weak policy outcomes. The results are supporting the idea that the failure of the public policy concerning this field is not only a matter of attitudinal issue but also a structural and cultural flaw.

These patterns were further enriched by the more qualitative conception of the patterns that had emotional and human dimension. According to the officers, they work in an environment that is characterized by a culture of under-investment, political meddling, and social resistance. Moral suffering was said to be rooted in much on a clash between their law and local standards. These stories demonstrate that institutional change cannot be conceptualized simply in administrative terms, but must also account for the emotional labor and psychological strength demanded of frontline operatives at the fault line of family protection. In this regard, Palestinian domestic violence policy is a trial both of human fortitude and bureaucratic efficacy.

The kind of analysis was anchored on the feminist, social learning theory and the conflict theory to offer a multi-planning theory of analysis results. Feminist theory provided a way to understand that gendered power shapes not only family members’ actions but also the behavior of state institutions, from private patriarchies to public policies. Social learning theory put the intergenerational transfer of violence into the spotlight, i.e., the cultural condoning of violence which plans into the acceptance across the generations. Conflict theory situated these dynamics in the larger context of Palestine’s structural inequalities, where occupation and economic uncertainty and political division add insult to social hierarchy. Combined, these frameworks emphasize that domestic violence reflects and resists systemic inequality a cycle propped up by weak governance and cultural inertia.

The study shows that the gap between the ambition and the capability of legislation continues to persist in policy terms. The officers noted that there were progressive policy frameworks in place (such as the draft Family Protection Law) but insisted that without being legally effected, resourced and with sustained inter-agency collaboration such a framework was purely symbolic. The evidence shows that the Palestinian protection regime was functioning in a scenario of the symbolic compliance whereby the institutions adhere to the international norms, but lacked the organizational power to enforce it. Lack of ratified Family Protection Law continued to create uncertainty under the law and reduce accountability, leaving victims to rely on informal dispute resolution methods in more remote or conservative areas.

Meanwhile, there exist obvious reform paths which are outlined in the study. The implementation of such a policy requires in parallel four lines of action: legislative, inter-agencies and institutions acquiring capacities that result in the transformation of the members of the organization. Passing the Family Protection Law is the vital first step, which provides a meaningful legal authority for protection and accountability. However, this must be accompanied by the introduction of changes in law—a system of standardized data, searchlights that support coordination between sectors councils and continuous training of professionals. Grassroots education against patriarchal narratives, and culture-clashing gestures of shared responsibility are also, at least, as crucial. A legal, institutional, and cultural change should send us to evolve to have real change at the end of the day that will be lasting.

There is also an important contribution for science and methodology in this study. It does so empirically—by falling back on the reality of the field, that is to say by grounding its analysis in practitioner voices: those who act as intermediaries translating policy into practice within messy sociopolitical environments. In theoretical terms, it contributes to the feminist and conflict traditions by highlighting how domestic violence is both an effect and a cause of institutional weakness. Methodologically, the mixed-methods approach offers a replicable protocol for studying social policy in zones of fragile or divided government. These contributions position the study in a new field of gender-sensitive policy analysis in conflict-affected countries.

However, there are also limitations of the analysis that could be the subject of future research. There could be bias in the reliance on self-reported information, and survivors’ point of view is missing. To be able to capture the entire policy ecosystem, future studies should include longitudinal and participative designs with the voices of both victims and communities added to those of practitioners. Comparative work with other Arab or post-conflict settings might also provide further insight into the role of sociopolitical structures through which gender-based violence and official response are accomplished.

In the end, the material presented has shown that responding to domestic violence in Palestine goes beyond technical reform; it necessitates structural, cultural and emotional change. The policies must go beyond compliance but they must have a demonstration of empathy, accountability and inclusion. Therefore, makers must not merely be defended as policy makers, but as the type of point of habitation, as the type of carrier of human dignity, in the limited domains of practice that they act. True protection is not laws and set-ups but moral will to maintain justice in families, as well as society. By linking empirical evidence and theoretical knowledge, this work contributes to an enhanced comprehension of social policy, symbolic politics and law as it may have moved from frameworks that symbolize equality and protection toward tools used to ‘do’ them in Palestine together with other contexts.

## Data Availability

The original contributions presented in the study are included in the article/supplementary material, further inquiries can be directed to the corresponding author.
